# The Cortical Lesion for Motor Aphasia

**Published:** 1909-05-15

**Authors:** 


### NEUROLOGY.

### THE CORTICAL LESION FOR MOTOR APHASIA.

Until a few years ago it seemed firmly established that the cortical motor area for speech in the human brain is in Broca's area in the operculum triangularis of the third left frontal convolution. Then Marie declared that this is all wrong, and that motor aphasia is due not to a lesion in Broca's area, but to affections of other parts of the brain. It will be a relief to those who do not like old-established ideas to be suddenly uprooted to hear of a case in which motor aphasia was caused by softening exactly localised to the foot of the third left frontal convolution.

It is clear that whether one destroys the root of a tree or cuts through the trunk close to the root the effect upon the branches will be the same; similarly, motor aphasia may be caused just as well by a lesion which destroys the fibres coming from Broca's area as by one which destroys Broca's area itself. The fact that a patient suffering from motor aphasia may die and exhibit a perfectly normal Broca's area is no proof that this is not the motor centre for speech in the cerebral cortex, for there may be a lesion in the white matter immediately beneath it, causing the same result as if the grey matter were itself destroyed.

The patient in question, as recorded by Chauffard and Rathery, was a woman, 61 years old when admitted to hospital. Two days previously she had hardly returned home from doing a little shopping when she suddenly felt ill and sat down in a chair. From that moment, without any apoplectic attack and without any loss of consciousness, she had been entirely unable to speak, though up to then her speech had been perfectly normal.

When seen in hospital it was obvious that there was facial paralysis below the eyelids on the right side, and a very slight diminution in power in the right arm and hand. Sensibility and all the ordinary reflexes were natural. There was neither albumin nor sugar in the urine. When she was spoken to it became clear that she was quite unable to reply. She made visible efforts to do so, but only succeeded in producing sounds that were unintelligible. She understood everything that was said to her perfectly, and at once performed any of the simple movements that she was asked to.

The diagnosis of motor aphasia seemed clear and beyond doubt. Further investigations were, however, impossible, for the malady progressed so rapidly that the patient quickly sank and died eight days later. There was a post-mortem examination, which entirely confirmed the older views as to speech localisation in Broca's area. On the surface of the left hemisphere, at the level of the opercula, particularly in that portion of the latter which is situated in front of the fissure of Rolando and at the base of the third frontal convolution, there was a red area well demarcated from the normal colour of the rest of the brain; the meninges at this spot were slightly raised by excess of blood-stained serous exudate beneath the pia mater; the brain tissue beneath was distinctly bulged and on section it was found to be softened and reddened—the softening being sharply restricted to the region mentioned.

Sections of the hemispheres at various levels showed that there was no lesion in the internal capsules, corpora striata, optic thalami, or white matter of the third frontal convolutions. At the level of the anterior convolution of the island of Reil there was a small focus of softening the size of a pea, and beneath the opercula in direct relation with the anterior convolution of the island of Reil there was another small focus of necrosis of similar size. Finally, close to the beginning of the second left frontal convolution there was a patch of red softening, of the area of a threepenny piece, similar in appearance to that in the third left frontal convolution, but less in extent. The main mass of softening, indeed, was confined to Broca's area.

When the left Sylvian fissure was opened up so as to expose the Sylvian artery, an ante-mortem thrombus was discovered at a bifurcation of the latter. Careful dissection showed that the branch that was blocked was precisely that which supplies the third left frontal convolution. Transverse sections showed that the clot was undoubtedly adherent to the vessel wall. Further examination showed that the original cause of the cerebral lesion was embolism from malignant endocarditis, the mitral valve being covered with exuberant vegetations. There were infarcts in the spleen and in the lungs.

The interest of the case lies mainly in the occurrence of motor aphasia from a lesion almost confined to Broca's area, notwithstanding recent expressions of opinion that Broca's area is not really the motor centre for intelligent speech.

